# Survival and Clinicopathological Significance of CD47 in Human Solid Tumors: An Updated Systematic Reviews and Meta‐Analysis

**DOI:** 10.1002/cnr2.70296

**Published:** 2025-08-05

**Authors:** Yongzhi Ye, Meiqiong Chen, Fada Ji, Suicai Mi, Zhixiong Chen, Xiaowei Wu, Qiurong He, Xiaodong Liu

**Affiliations:** ^1^ Department of General Surgery Xiamen Hospital of Traditional Chinese Medicine Xiamen People's Republic of China; ^2^ Department of Operation Management Women and Children’s Hosipital, School and Medicine, Xiamen University Xiamen People's Republic of China; ^3^ Department of Oncology Xiamen Hospital of Traditional Chinese Medicine Xiamen People's Republic of China

**Keywords:** CD47, meta‐analysis, prognosis, tumor

## Abstract

**Background:**

High expression levels of cluster of differentiation 47 (CD47) have been recognized as poor survival in several different cancers. Nevertheless, the significance of CD47 in patients with solid tumors remains controversial.

**Aims:**

The objective of this study is to elucidate whether elevated CD47 expression independently predicts a poor prognosis across solid tumors through pooled survival and clinicopathological analyses.

**Methods and Results:**

This meta‐analysis was based on a search of PubMed, Embase, and Web of Science databases to obtain 20 eligible published studies (totaling 4019 patients) between January 2018 and January 2024. The combined hazard ratios (HRs) for overall survival (OS) were evaluated, and the HRs for relapse‐free survival (RFS), progression‐free survival (PFS), and disease‐free survival (DFS), as well as odds ratios for clinicopathological data, were also respectively combined. The data obtained from these studies were extracted from these published studies and analyzed. This study suggested that CD47 overexpression was related to shorter OS times in human solid tumors, with a combined HR for OS (according to the univariate analysis) of HR = 1.63 (95% confidence intervals,[ 95% CIs]: 1.45–1.83; *p* < 0.00001), and a pooled HR for OS (according to the multivariate analysis) of HR = 2.02 (95% CI: 1.43–2.84; *p* < 0.0001). The subgroup analysis revealed that CD47 overexpression was related to inferior OS rates according to country, cancer type, sample size, analysis type, and the method via which the HR value was obtained (i.e., reported or extracted; *p* < 0.05); in addition, a high expression level of CD47 was also a predictor of poor DFS, PFS, and RFS rates (*p* < 0.00001). Certain factors, such as age (≥ 60 years old), lymph node metastasis, TNM staging, differentiation type, and tumor recurrence, resulted in an upregulation of CD47 (*p* < 0.05).

**Conclusion:**

This meta‐analysis indicates that CD47 overexpression is significantly associated with poor clinical outcomes, advanced clinical stages, and poor differentiation in solid tumor patients, particularly, in cases involving tumors in the digestive and respiratory systems. These findings suggest that CD47 could serve as a valuable prognostic biomarker and therapeutic target.

**Abbreviations**
BC: breast cancer
CD47: cluster of differentiation 47
cHL: classical Hodgkin's lymphoma
CRC: colorectal cancer
DFS: disease free survival
DLBCL: diffuse large B cell lymphoma
EC: endometrial carcinoma
EFS: event free survival
GC: gastric cancer
HR: hazard ratio
LNET: lung neuroendocrine tumors
LUAD: lung adenocarcinoma
LUSC: lung squamous cell carcinoma
MIBC: muscle invasive bladder cancer
NMIBC: nonmuscle invasive bladder cancer
NOS: Newcastle–Ottawa scale
NPC: nasopharyngeal carcinoma
NR: not reported
OR: odds ratio
OS: overall survival
PFS: progression free survival
PSC: pulmonary sarcomatous carcinoma
RFS: recurrence free survival
SCLC: small cell lung cancer
SI: staining index
TPS: tumor proportion score

## 1 Introduction

The global burden of cancer, particularly, solid tumors, represents a significant public health challenge, characterized by high incidence and mortality rates. As evidenced by recent statistics, cancer remains a leading cause of mortality on a global scale, underscoring the urgent need to elucidate its underlying mechanisms and potential therapeutic targets [1]. The identification of biomarkers associated with solid tumors has emerged as a critical area of research, as these biomarkers can significantly enhance clinical diagnosis and treatment strategies [2]. Notably, the search for biomarkers that can predict tumor prognosis is well‐established in the literature, indicating that such biomarkers can improve treatment efficacy and patient survival rates [3]. Therefore, the exploration of biomarkers linked to solid tumors holds considerable promise for advancing cancer prognosis and patient outcomes.

Among the various biomarkers that have been the subject of study, CD47 has attracted considerable attention due to its function as a highly glycosylated immunoglobulin that is typically expressed at low levels in normal cells [4]. The interaction between CD47 and signal regulatory protein α (SIRPα), which is primarily expressed by macrophages and dendritic cells, is of great importance in the context of immune evasion. Upon binding to SIRPα, CD47 transmits an inhibitory signal that prevents phagocytosis, thereby allowing tumor cells to evade immune surveillance [5]. This mechanism of immune evasion is a significant cause for concern, as it may facilitate the growth and metastasis of various cancers, thereby contributing to poor clinical outcomes [6].

A substantial body of literature demonstrates a correlation between the overexpression of CD47 in a multitude of solid tumors and a reduction in patient survival rates. For example, studies have documented high CD47 expression in esophageal cancer [7], gastric cancer [5, 8], colorectal cancer [9], hepatocellular carcinoma [10], lung cancer [4, 6, 11], breast cancer [12], endometrial cancer [13, 14], kidney cancer [15], and nasopharyngeal cancer [16]. However, the prognostic significance of CD47 is not universally accepted, as some studies have reported contradictory findings. For example, Lang et al. [17] found that CD47 did not demonstrate prognostic significance in lung cancer, a conclusion echoed in a study on hepatocellular carcinoma [18]. This inconsistency highlights the need for further investigation into the role of CD47 as a prognostic biomarker in solid tumors.

In light of the inconsistencies in the evidence pertaining to the prognostic value of CD47, this meta‐analysis seeks to elucidate its role as a biomarker in solid tumors. By conducting a comprehensive review of the latest literature, our objective is to gain insight into the role of CD47 in various malignancies and to evaluate its potential clinical applications. In conclusion, our objective is to deepen the comprehension of CD4's implications for patient outcomes and to provide guidance for future therapeutic strategies targeting this pathway. The findings from this analysis have the potential to facilitate enhanced prognostic assessments and treatment modalities, thereby contributing to more effective management of solid tumors.

## 2 Methods

### 2.1 Search Strategy

Systematically searched literatures between January 2018 and January 2024 in PubMed, Embase, and Web of Science databases. The search included the following terms: “CD47 antigen*,” “Thrombospondin‐1 receptor,” “IAP‐50 antigen,” “tumor*,” “neoplasm*,” “cancer*,” “neoplasia*,” “malignancy,” “prognosis,” “survival,” and the studies were limited to patients with solid tumors, with manually screening of all potential studies in the included literatures. Each potential study for inclusion was assessed and identified by two researchers (Y.Y. and M.C.), and any ambiguities were adjudicated by the corresponding author (X.L.).

### 2.2 Inclusion Criteria

(i) The study must investigate the prognostic association between CD47 and OS and/or DFS, PFS, and RFS; (ii) the follow‐up period must be 60 months or above; (iii) only studies written in English will be considered.

### 2.3 Exclusion Criteria

(i) The studies included animal experiments or did not feature human solid tumors; (ii) the study contained insufficient data to obtain the HR value; (iii) the study was a duplicate report or an other type, such as reviews and case reports.

### 2.4 Data and Quality

The data set included the surname of the first author, the type of cancer, the number of patients, the duration of the follow‐up period, the inspection method, the value of the cut‐off, the percentage of CD47 expression, the results of the survival analysis, the HR (obtained), as well as the clinicopathological characteristics of solid tumor patients (including age, sex, lymph node and distant metastasis, tumor differentiation, TNM staging, tumor recurrence, and smoke exposure). The relationship between smoke exposure and CD47 expression in diverse lung cancer subtypes represents a pivotal avenue of investigation, particularly, in light of its implications for prognosis and treatment strategies. The data regarding the hazard ratio (HR) were either obtained from the literature or calculated from Kaplan–Meier (K‐M) curves. The quality of each included literature source was evaluated using the Newcastle–Ottawa Scale (NOS) [19]. The data extraction was carried out in collaboration between two researchers (Y.Y. and M.C.), and any ambiguities were adjudicated by the corresponding author (X.L.).

Overall survival (OS) was the time of the last available follow‐up or death. DFS, PFS, and RFS were defined as the time of metastasis or recurrence. Disease‐specific survival (DSS) data included the data for DFS, PFS, and RFS. Finally, event‐free survival (EFS) represented the time of tumor relapse or progression, first‐line treatment failure, or death.

### 2.5 Statistical Analysis

Typically, statistical variables were extracted directly from the literature. Subsequently, prognostic data for studies including only K‐M curves were extracted using Engauge Digitizer v.4.1 and the Tierney method [20]. Cochran's *Q* test and Higgins's *I* ^2^ statistic were employed to quantify the heterogeneity of individual HRs [21, 22]. A further subgroup analysis was conducted to account for the observed heterogeneity, and a sensitivity analysis was employed to assess the robustness of the included studies. Additionally, odds ratios (ORs) were calculated to determine the relationship between CD47 expression and clinicopathological features. Funnel plots were employed in conjunction with Begg's and Egger's tests to assess the potential for publication bias. The results were considered to indicate significant bias if the *p*‐values were less than 0.05 [23]. Finally, the statistical analysis was conducted using Review Manager 5.3 and Stata v.14.0 software (Stata Corp LP).

## 3 Results

**FIGURE 1:**
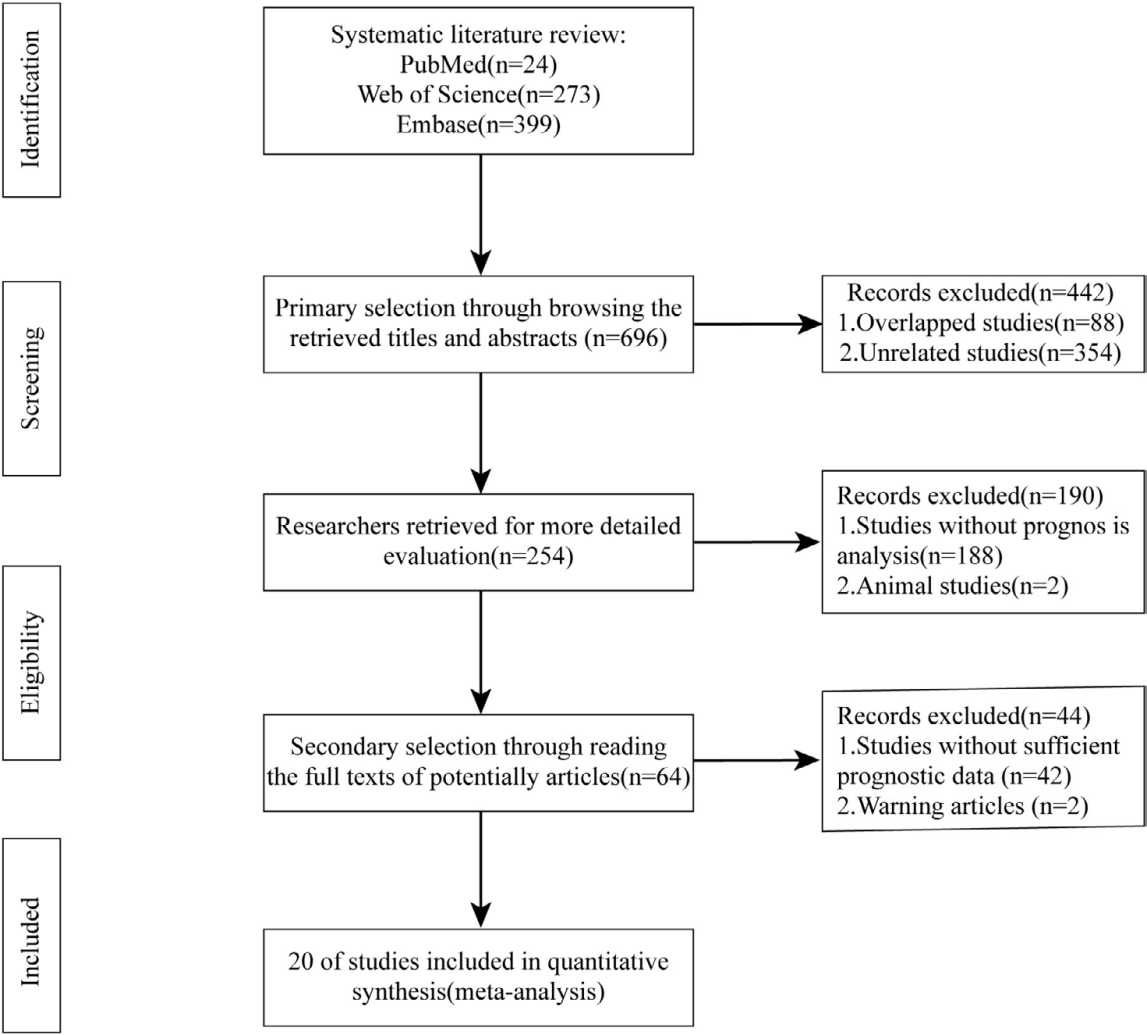
Flowchart of the search strategy.

The preliminary search identified 696 associated studies. Twenty studies in total were finally included, totaling 4019 cases (Figure 1). Table 1 showed the detailed baseline characteristics of the 20 eligible studies. The search scope included seven countries (namely, China, Japan, South Korea, Mexico, Norway, Sweden, and Turkey), involving studies published between January 2018 and January 2024. A total of 11 different cancer types were included, that is, lung cancer (*n* = 6), gastric cancer (*n* = 2), bladder cancer (*n* = 1), lymphoma (*n* = 2), hepatocellular carcinoma (*n* = 2), endometrial cancer (*n* = 2), colorectal cancer (*n* = 1), nasopharyngeal carcinoma (*n* = 1), breast cancer (*n* = 1), kidney cancer (*n* = 1) and esophageal cancer (*n* = 1). Among them, 18 studies provided OS (univariate analysis) data, 8 studies included OS (multivariate analysis) data, and 12 studies presented DSS data (3 DFS, 4 PFS, and 5 RFS). The detection methods for CD47 vary across different studies, resulting in inconsistent threshold values for CD47.

**TABLE 1:** Baseline characteristics of the included studies.

Paper reference	Country	Cancer type	Sample size	Follow‐up (months)	TNM stage	Detection method	CD47 (+) (*N*, %)	Survival analysis	HR (obtain)
Akane et al. [9]	Japan	CRC	269	> 80	NR	IHC (NR)	95 (35.3)	OS (U, M)	R/E
Arrieta et al. [3]	Mexico	NSCLC	169	> 60	IIIBIV	IHC (Score ≥ 150)	79 (46.7)	OS (U)	E
Chen et al. [10]	China	HCC	390	> 100	IIV	IHC (H‐Score ≥ 11.5)	193 (49.5)	OS (U, M)/RFS (U, M)	R/E
Chen et al. [8]	China	GC	268	6080	IIV	IHC (Score ≥ 2)	122 (45.5)	OS (U)	R
Chen et al. [12]	Norway	BC	282	> 150	NR	IHC (SI ≥ 6)	NR	RFS (U, M)	R
Gholiha et al. [24]	Sweden	cHL	178	> 180	NR	IHC (Score ≥ 8)	64 (36.0)	OS (U, M)/EFS (U, M)	R/E
Jiang et al. [15]	China	ccRCC	235	120 144	IIV	IHC (Score ≥ 90)	118 (50.2)	OS (U, M)/RFS (U, M)	R/E
Kazama et al. [25]	Japan	DLBCL	120	96 108	NR	IHC (NR)	71 (59.2)	OS (U, M)/PFS (U, M)	R
Kim et al. [18]	Korea	HCC	166	240 288	IIV	IHC (Score ≥ 10)	36 (21.7)	OS (U)/RFS (U)	E
Lang et al. [17]	Sweden	SCLC	104	200 300	IIV	IHC (≥ 10%)	88 (84.6)	OS (U)/DFS (U)	R
Mario et al. [2]	Mexico	LNET	51	100 120	IIIBIV	IHC (H‐Score ≥ 30)	14 (27.5)	OS (U)/PFS (U)	R
Sercan et al. [13]	Turkey	EC	165	> 125	IIV	IHC (Score ≥ 2)	101 (61.2)	OS (U, M)/PFS (U, M)	R/E
Wang et al. [26]	China	NMIBC	169	> 60	NR	IHC (Score > 4)	118 (69.8)	RFS (U, M)	R/E
Wang et al. [7]	China	ESCC	148	> 120	IIV	IHC (H‐Score ≥ 125)	57 (43.2)	OS (U, M)/PFS (U, M)	R
Wang et al. [16]	China	NPC	66	100 120	IIIVA	IHC (TPS > 10%)	37 (56.1)	OS (U)/DFS (U, M)	R/E
Xu et al. [4]	China	NSCLC	191	> 120	I, III	IHC (Score ≥ 2)	63 (33.0)	DFS (U, M)	R
Yang et al. [11]	China	PSC	148	> 100	IIV	IHC (TPS ≥ 10%)	78 (52.7)	OS (U)	R
Yang et al. [6]	China	LUSC	190	> 140	IIV	IHC (TPS ≥ 5%)	124 (65.3)	OS (U)	R
Yang et al. [6]	China	LUAD	240	> 125	IIV	IHC (TPS ≥ 5%)	172 (71.7)	OS (U)	R
Yang et al. [14]	China	EC	344	> 200	III	IHC (NR)	259 (75.3)	OS (U)	R
Zhou et al. [5]	China	GC	126	80 100	IIII	IHC (Score > 2)	83 (65.9)	OS (U, M)	R/E

Abbreviations: BC, breast cancer; ccRCC, clear cell renal cell carcinoma; cHL, classical Hodgkin's lymphoma; CRC, colorectal cancer; DFS, disease‐free survival; DLBCL, diffuse large Bcell lymphoma; E, extracted; EC, endometrial carcinoma; EFS, event‐free survival; ESCC, esophageal squamous cell carcinoma; GC, gastric cancer; HCC, hepatocellular carcinoma; HR, hazard ratio; IHC, immunohistochemistry; LNET, lung neuroendocrine tumors; LUAD, lung adenocarcinoma; LUSC, lung squamous cell carcinoma; M, multivariate analysis; NMIBC, nonmuscleinvasive bladder cancer; NPC, nasopharyngeal carcinoma; NR, not reported; NSCLC, nonsmall cell lung cancer; OS, overall survival; PFS, progression‐free survival; PSC, pulmonary sarcomatous carcinoma; R, reported; RFS, recurrence‐free survival; SCLC, small cell lung cancer; SI, staining index; TPS, tumor proportion score; U, univariate analysis.

### 3.1 CD47 Expression and OS

**FIGURE 2:**
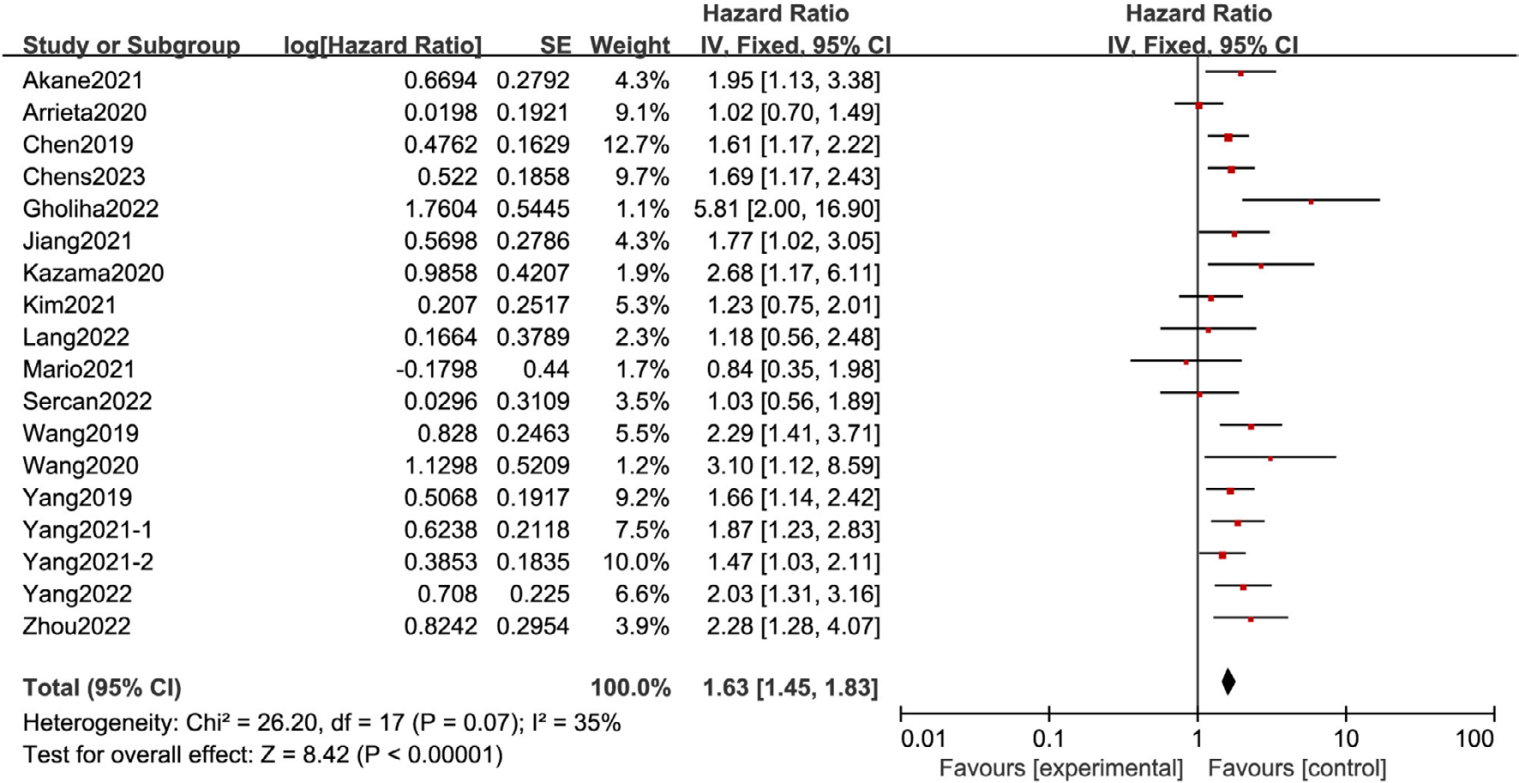
The relation between CD47 overexpression and OS (univariable analysis). OS, overall survival [2, 3, 5, 6, 7, 8, 9, 10, 11, 13, 14, 15, 16, 17, 18, 24, 25].

**FIGURE 3:**
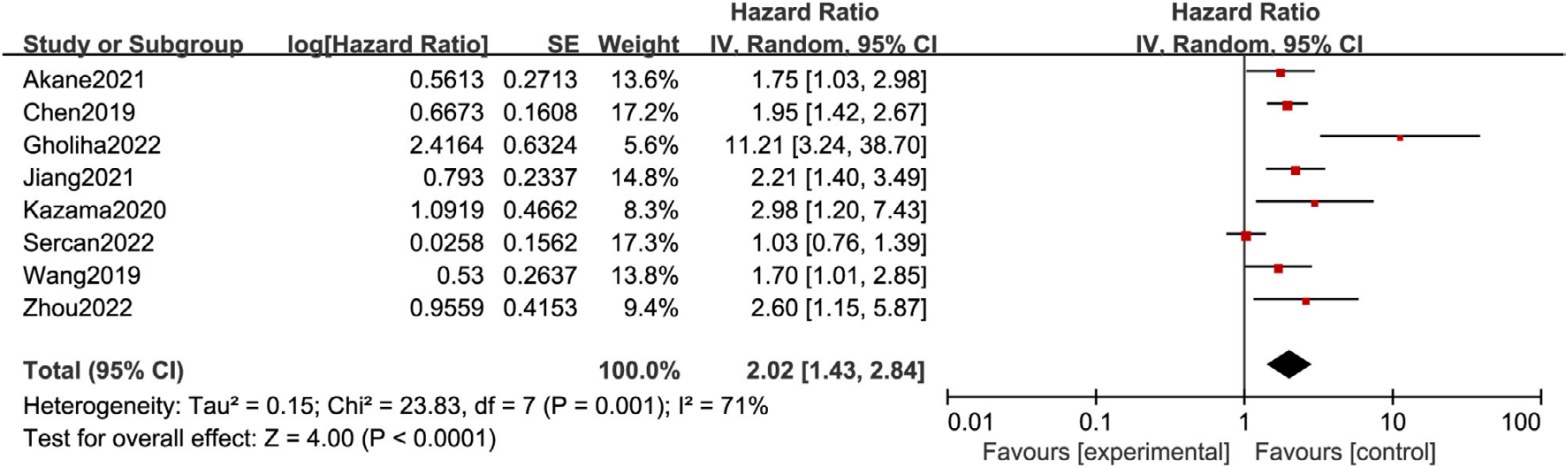
The relation between CD47 overexpression and OS (multivariable analysis). OS, overall survival [5, 7, 9, 10, 13, 15, 24, 25].

**FIGURE 4:**
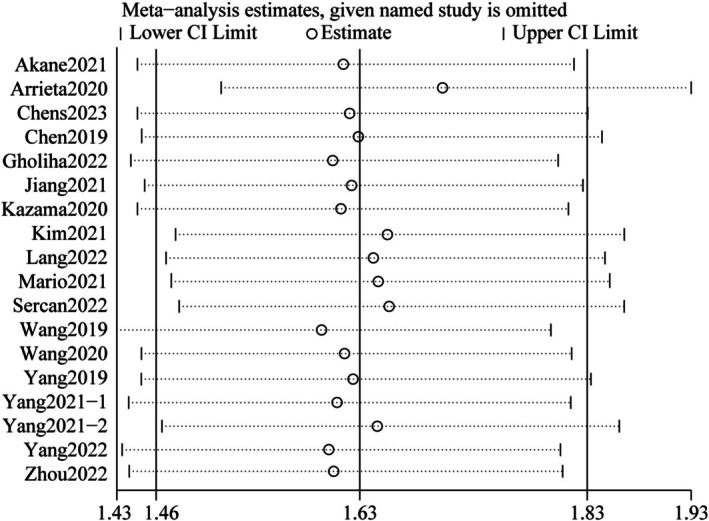
Sensitivity analysis of OS. OS, overall survival [2, 3, 5, 6, 7, 8, 9, 10, 11, 13, 14, 15, 16, 17, 18, 24, 25].

**FIGURE 5:**
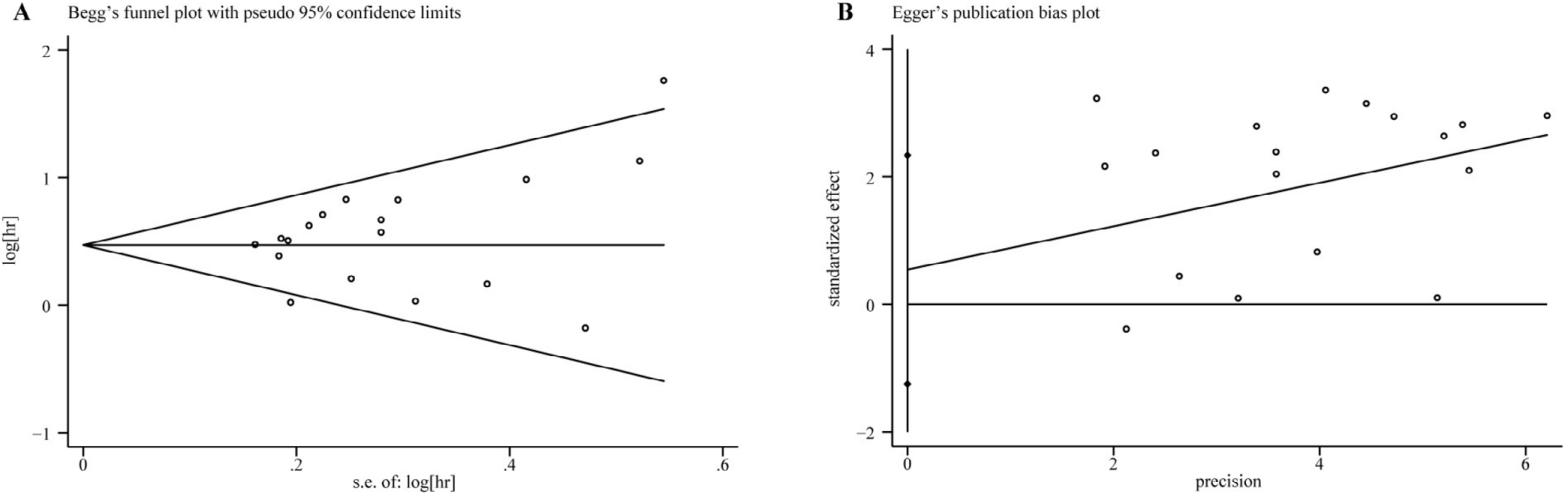
(A) Begg's funnel plot for CD47 expression and OS, and (B) Egger's publication bias plot for CD47 expression and OS. OS, overall survival [2, 3, 5, 6, 7, 8, 9, 10, 11, 13, 14, 15, 16, 17, 18, 24, 25].

**FIGURE A1:**
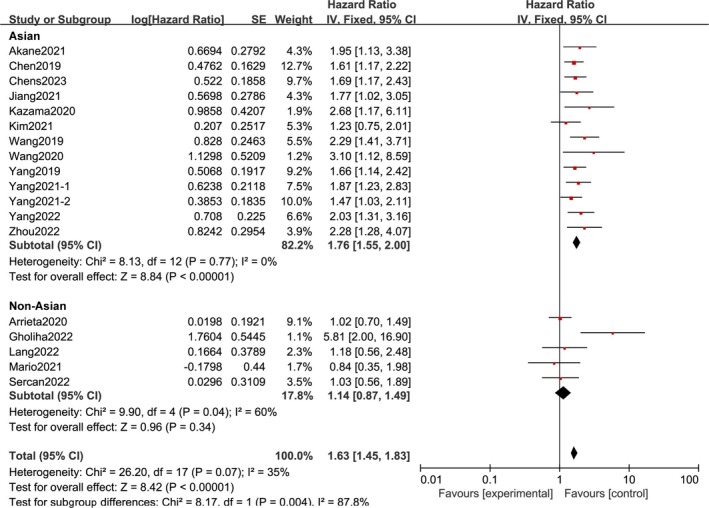
Subgroup analysis for OS (univariable analysis) according to country. OS, overall survival. [5, 6, 7, 8, 9, 10, 11, 14, 15, 16, 18, 25] versus [2, 3, 13, 17, 24].

**FIGURE A2:**
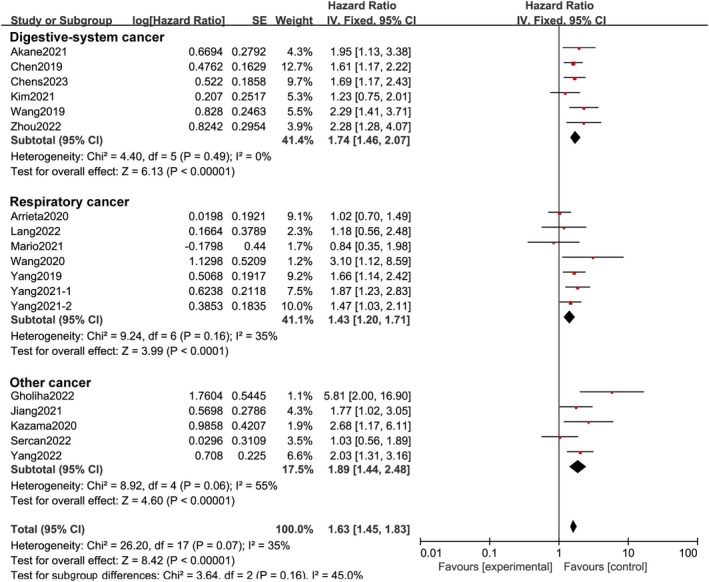
Subgroup analysis for OS (univariable analysis) according to cancer type. OS, overall survival. [5, 7, 8, 9, 10, 18] versus [2, 3, 6, 11, 16, 17] versus [13, 14, 15, 24, 25].

**FIGURE A3:**
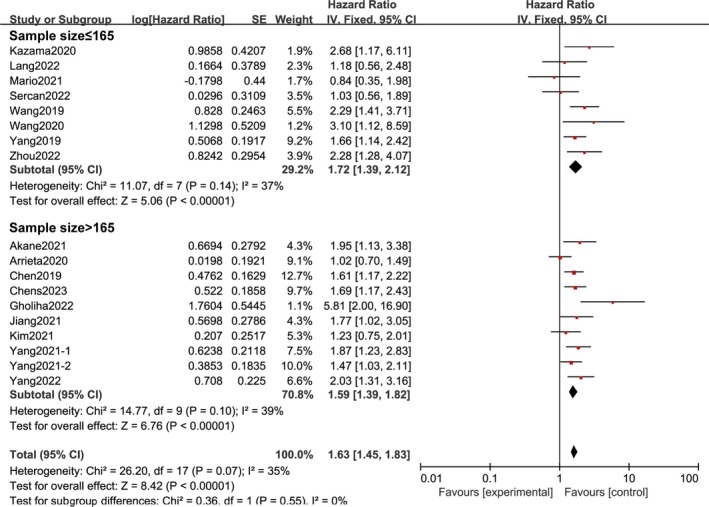
Subgroup analysis for OS (univariable analysis) according to sample size. OS, overall survival. [2, 5, 7, 11, 13, 16, 17, 25] versus [3, 6, 8, 9, 10, 14, 15, 18, 24].

**FIGURE A4:**
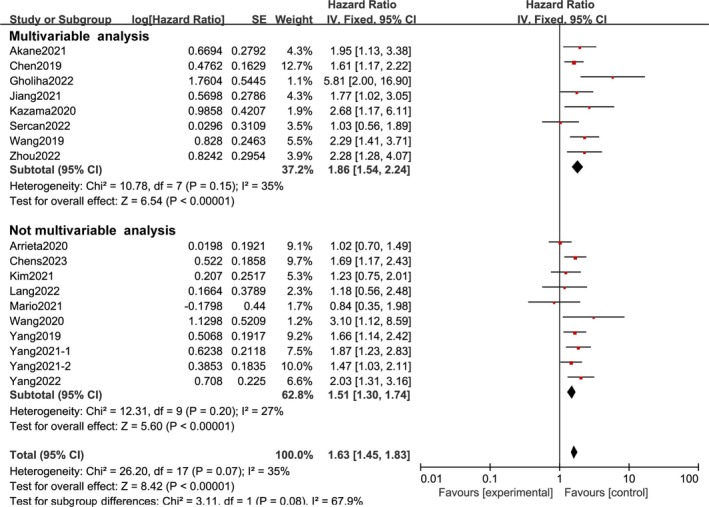
Subgroup analysis for OS (univariable analysis) according to analysis type. OS, overall survival. [5, 7, 9, 10, 13, 15, 24, 25] versus [2, 3, 6, 8, 11, 14, 16, 17, 18].

**FIGURE A5:**
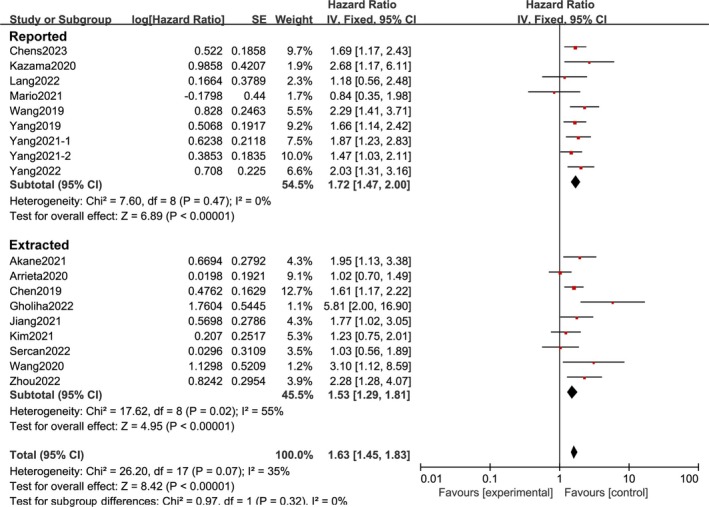
Subgroup analysis for OS (univariable analysis) according to HR‐obtained method. OS, overall survival. [2, 6, 7, 8, 11, 14, 17, 25] versus [3, 5, 9, 10, 13, 15, 16, 18, 24].

**FIGURE A6:**
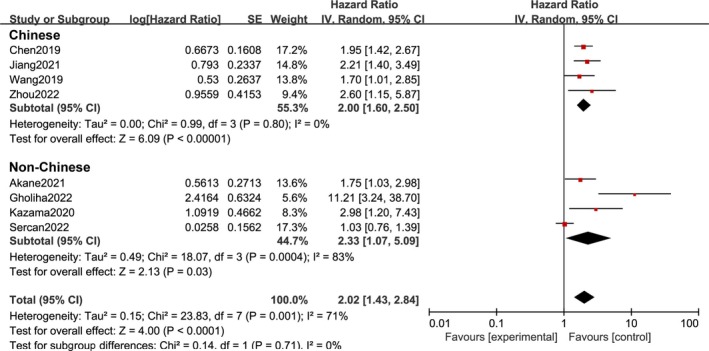
Subgroup analysis for OS (multivariable analysis) according to country. OS, overall survival. [5, 7, 10, 15] versus [9, 13, 24, 25].

**FIGURE A7:**
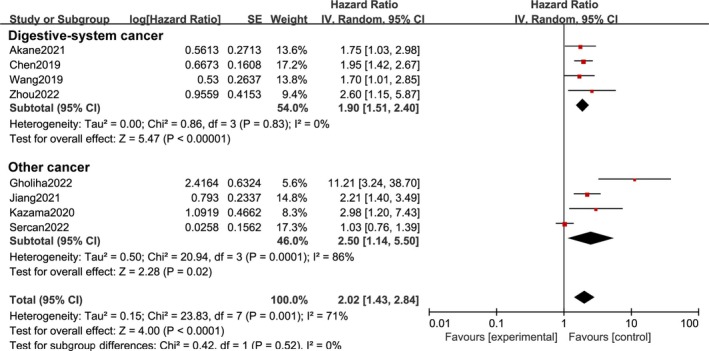
Subgroup analysis for OS (multivariable analysis) according to cancer type. OS, overall survival. [5, 7, 9, 10] versus [13, 15, 24, 25].

**FIGURE A8:**
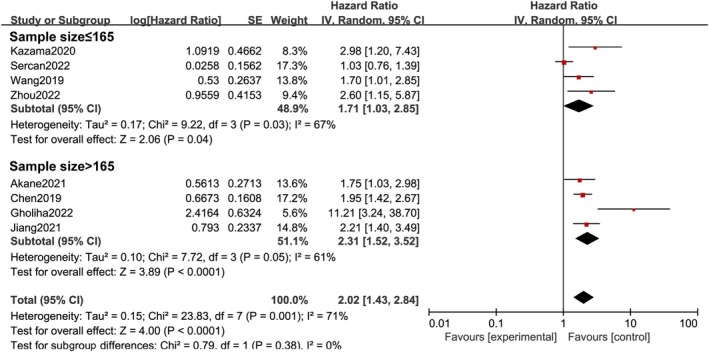
Subgroup analysis for OS (multivariable analysis) according to sample size. OS, overall survival. [5, 7, 13, 25] versus [9, 10, 15, 24].

The findings indicated that the high expression of CD47 is significantly related to inferior survival in patients with some solid tumors. The pooled HR (HR = 1.63; 95% CI: 1.45–1.83; *p* < 0.00001) of OS (univariate analysis) showed no significant heterogeneity (Figure 2). Among them, eight studies also conducted multivariate analysis of OS, and the pooled HR (HR = 2.02; 95% CI: 1.43–2.84, *p* < 0.0001) of OS (multivariate analysis) suggested heterogeneity (*p* = 0.001; *I* ^2^ = 71%) (Figure 3). To further explore heterogeneity, subgroup analysis for OS (univariate analysis) was conducted according to the factors, such as country, sample size, tumor type, analysis, and HR‐obtained method. The subgroup analysis revealed that high CD47 expression was related to poorer OS in digestive system tumors (HR = 1.74; 95% CI: 1.46–2.07; *p* < 0.00001), respiratory system tumors (HR = 1.43; 95% CI: 1.20–1.71; *p* < 0.0001) and other system tumors (HR = 1.89; 95% CI: 1.44–2.48; *p* < 0.00001), however, it is important to note the heterogeneity that exists within the subgroup of other system tumors. The combined HR values (95% CI) for the remaining subgroups (with the exception of the non‐Asian one) were also > 1.00 (*p* < 0.05). Aside from the non‐Asian and HR‐extracted subgroup, however, no significant heterogeneity was observed (Table 2 and Figures A1, A2, A3, A4, A5). The heterogeneity of OS in multivariate analysis was also performed based on the country, tumor type, and sample size. The results suggested that high CD47 expression was also associated with an adverse prognosis for digestive system tumors (HR = 1.90; 95% CI: 1.51–2.40; *p* < 0.00001). The combined HRs (95% CI) for the country and sample size subgroups were > 1.00 (*p* < 0.05), respectively, although with heterogeneity in both groups (Table 3 and Figures A6, A7, A8). Subsequently, a sensitivity analysis for removing any single study was found to have no significant impact on the combined HRs (Figure 4). The average NOS score of total of 20 studies was 7.6, suggesting reliability (Table 4). Funnel plot with Begg's test (*p* = 0.130) and Egger's test (*p* = 0.239) showed no significant publication bias in these analyses (Figure 5).

**TABLE 2:** Subgroup analyses for OS (univariable analysis).

Subgroup	No. of studies	No. of patients	Pooled HR (95% CI)	*p*	Heterogeneity	
					*I* ^2^ (%)	*p*
Overall survival	18	3377	1.63 (1.45–1.83)	< 0.00001	35	0.07
Country						
Asian	13	2710	1.76 (1.55–2.00)	< 0.00001	0	0.77
Non‐Asian	5	667	1.14 (0.87–1.49)	0.34	60	0.04
Cancer type						
Digestive‐system cancer	6	1367	1.74 (1.46–2.07)	< 0.00001	0	0.49
Respiratory cancer	7	968	1.43 (1.20–1.71)	< 0.0001	35	0.16
Others	5	1042	1.89 (1.44–2.48)	< 0.00001	55	0.06
Sample size						
≤ 165	8	928	1.72 (1.39–2.12)	< 0.00001	37	0.10
> 165	10	2449	1.59 (1.39–1.82)	< 0.00001	39	0.10
Analysis type						
Multivariate	8	1631	1.86 (1.54–2.24)	< 0.00001	35	0.15
Nonmultivariate	10	1746	1.51 (1.30–1.74)	< 0.00001	27	0.20
HRobtained method						
Reported	9	1613	1.72 (1.47–2.00)	< 0.00001	0	0.47
Extracted	9	1764	1.53 (1.29–1.81)	< 0.00001	55	0.02

Abbreviations: HR, hazard ratio; OS, overall survival.

**TABLE 3:** Subgroup analyses for OS (multivariate analysis).

Subgroup	No. of studies	Pooled HR (95% CI)	*p*	Model	Heterogeneity	
					*I* ^2^ (%)	*p*
Country						
Chinese	4	2.00 (1.60–2.50)	< 0.00001	Fixed	0	0.80
Non‐Chinese	4	2.33 (1.07–5.09)	0.03	Random	83	0.0004
Cancer type						
Digestive‐system cancer	4	1.90 (1.51–2.40)	< 0.00001	Fixed	0	0.83
Others	4	2.50 (1.14–5.50)	0.02	Random	86	0.0001
Sample size						
≤ 165	4	1.71 (1.03–2.85)	0.04	Random	67	0.03
> 165	4	2.31 (1.52–3.52)	< 0.0001	Random	61	0.05

Abbreviations: HR, hazard ratio; OS, overall survival.

**TABLE 4:** Quality assessment of eligible studies with Newcastle–Ottawa Scale.

Article	Year	Selection	Comparability	Outcome	NOS
Akane et al. [9]	2021	★★★	★★	★★	7
Arrieta et al. [3]	2020	★★★	★★	★★	7
Chen et al. [8]	2023	★★★	★★	★★★	8
Chen et al. [10]	2019	★★★	★★	★★	7
Chen et al. [12]	2023	★★★★	★★	★★★	9
Gholiha et al. [24]	2022	★★★	★★	★★★	8
Jiang et al. [15]	2021	★★★	★★	★★★	8
Kazama et al. [25]	2020	★★★	★★	★★	7
Kim et al. [18]	2021	★★★	★★	★★	7
Lang et al. [17]	2022	★★★	★★	★★★	8
Mario et al. [2]	2021	★★★	★★	★★	7
Sercan et al. [13]	2022	★★★	★★	★★★	8
Wang et al. [26]	2018	★★★	★★	★★★	8
Wang et al. [7]	2019	★★★	★★	★★	7
Wang et al. [16]	2020	★★★	★★	★★	7
Xu et al. [4]	2020	★★★	★★	★★	7
Yang et al. [11]	2019	★★★	★★	★★★	8
Yang et al. [6]	2021	★★★	★★	★★★	8
Yang et al. [14]	2022	★★★	★★	★★	7
Zhou et al. [5]	2022	★★★	★★	★★★	8

*Note:* Each star flags 1 quality domain met.

Abbreviation: NOS, Newcastle–Ottawa scale.

### 3.2 CD47 Expression and DSS

**FIGURE 6:**
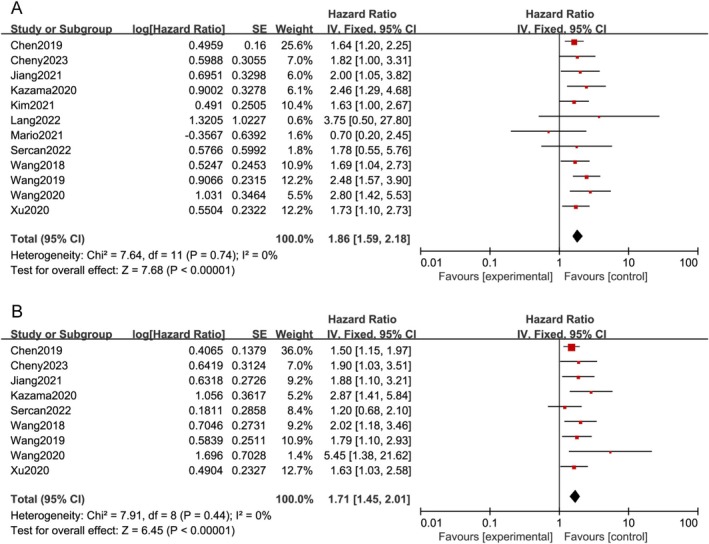
(A) The relation between CD47 overexpression and DSS (univariable analysis) [2, 4, 7, 10, 12, 13, 15, 16, 17, 18, 25, 26], and (B) the relation between CD47 overexpression and DSS (multivariable analysis) [4, 7, 10, 12, 13, 15, 16, 25, 26]. DSS, disease‐specific survival.

In this meta‐analysis, 12 studies involving 2087 patients were found to report that CD47 overexpression was related to poor DFS, PFS, and RFS (HR = 1.86; 95% CI: 1.59–2.18; *p* < 0.00001), and heterogeneity was not identified (I^2^ = 0.00%; *p* = 0.74) (Figure 6A). Among them, a multivariate analysis for DSS was also performed for nine studies comprising 1766 patients. The pooled HRs estimated by the fixed‐effects model (*I* ^2^ = 0.00%; *p* = 0.44), which also suggested that CD47 overexpression was positively correlated with inferior DSS (HR = 1.71; 95% CI: 1.45–2.01; *p* < 0.00001) (Figure 6B).

### 3.3 CD47 Expression and Clinicopathologic Features

**FIGURE A9:**
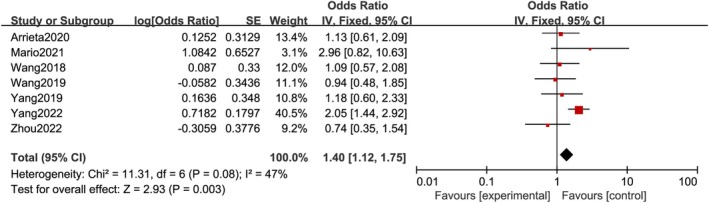
The odds ratio for CD47 overexpression and age (≥ 60 vs. 60 years). [2, 3, 5, 7, 11, 14, 26].

**FIGURE A10:**
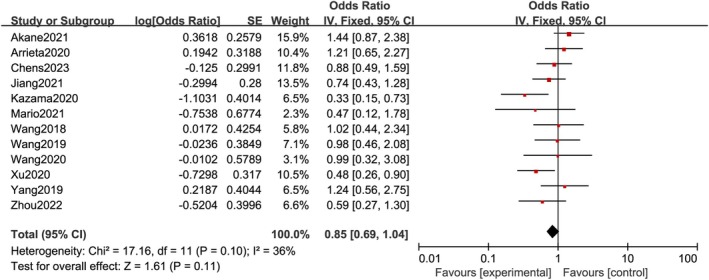
The odds ratio for CD47 overexpression and sex (male vs. female). [2, 3, 4, 5, 7, 8, 9, 11, 15, 16, 25, 26].

**FIGURE A11:**
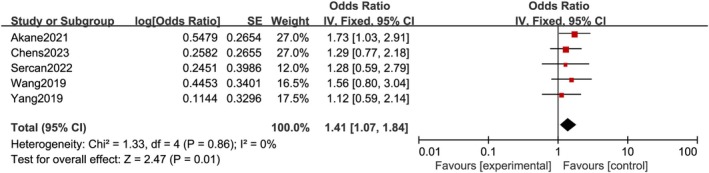
The odds ratio for CD47 overexpression and lymph node metastasis (positive vs. negative) [7, 8, 9, 11, 13].

**FIGURE A12:**
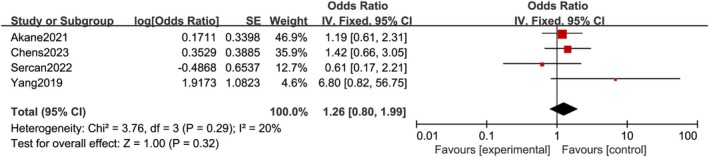
The odds ratio for CD47 overexpression and distant metastasis (positive vs. negative) [8, 9, 11, 13].

**FIGURE A13:**
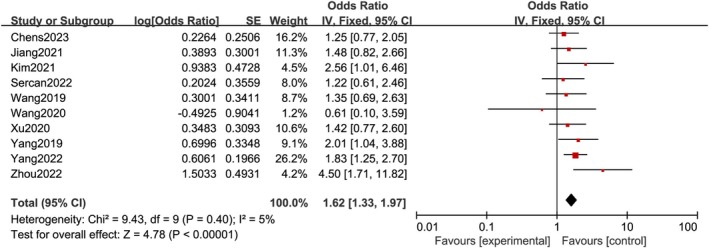
The odds ratio for CD47 overexpression and TNM staging (III and IV vs. I and II) [4, 5, 7, 8, 11, 13, 14, 15, 16, 18].

**FIGURE A14:**
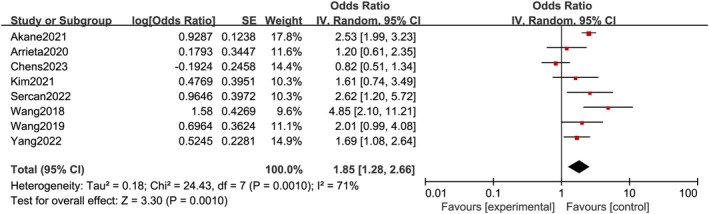
The odds ratio for CD47 overexpression and tumor differentiation (poorly or moderately vs. well‐differentiated) [3, 7, 8, 9, 13, 14, 18, 26].

**FIGURE A15:**
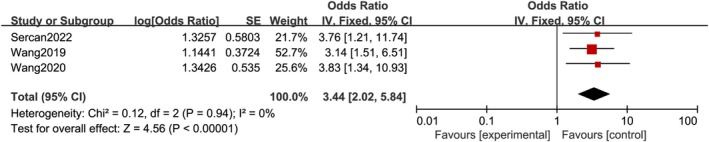
The odds ratio for CD47 overexpression and tumor recurrence (positive vs. negative) [7, 13, 16].

**FIGURE A16:**
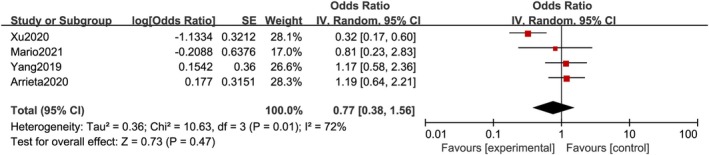
The odds ratio for CD47 overexpression and smoke exposure (positive vs. negative) [2, 3, 4, 11].

Table 5 and Figures A9, A10, A11, A12, A13, A14, A15 illustrate the combined ORs (95% confidence intervals) of clinicopathologic features. No significant correlation was observed between CD47 expression and sex or distant metastasis (*p* > 0.05). However, a significant association was identified between CD47 expression and age (≥ 60 years old), TNM staging, lymph node metastasis, tumor differentiation, and tumor recurrence (*p* < 0.05). Additionally, no significant correlation was identified between CD47 expression and smoke exposure (Please refer to Table S1 and Figure A16 for additional details.)

**TABLE 5:** Association of CD47 expression and clinicopathological parameters.

Clinicopathological parameter	No. of studies	No. of patients	OR (95% CI)	*p*	Heterogeneity	
					*I* ^2^ (%)	*p*
Age (≥ 60 years vs. < 60 years)	7	1155	1.40 (1.12–1.75)	0.003	47	0.08
Sex (male vs. female)	12	1960	0.85 (0.69–1.04)	0.11	36	0.10
Lymph node metastasis (+ vs. −)	5	998	1.41 (1.07–1.84)	0.01	0	0.86
Distant metastasis (+ vs. −)	4	850	1.26 (0.80–1.99)	0.32	20	0.29
TNM stage (III + IV vs. I + II)	10	1857	1.62 (1.33–1.97)		5	0.40
Differentiation (poor/moderately vs. well‐differentiated)	8	1698	1.85 (1.28–2.66)	0.001	71	0.001
Recurrence (+ vs. −)	3	379	3.44 (2.02–5.84)	< 0.00001	0	0.94

Abbreviation: OR, odds ratio.

## 4 Discussion

The role of CD47 in human solid tumors has recently received increased attention due to its association with a poor prognosis across various malignancies. The present study offers a comprehensive meta‐analysis of CD47 expression across 11 cancer types, involving 4019 patients from seven countries. The findings of this study indicate that high CD47 expression is significantly associated with inferior OS and DSS in various solid tumors. The pooled HRs for OS and DSS were 1.63 (95% CI: 1.45–1.83) and 1.86 (95% CI: 1.59–2.18), respectively. The subgroup analysis indicated that elevated CD47 expression was significantly associated with reduced OS in both digestive system and respiratory system tumors. These findings build upon the observations of previous studies, including the meta‐analysis by Zhao et al. [27], which encompassed a broader spectrum of cancers but reported analogous trends in OS (HR = 1.49; 95% CI: 1.36–1.62). The current results are consistent with those reported by Son et al. [28], who observed a correlation between CD47 expression and poor patient survival outcomes. Our analysis also highlights significant correlations between CD47 expression and clinicopathologic features, including age (≥ 60 years old), TNM staging, lymph node metastasis, tumor differentiation, and tumor recurrence, providing new insights into the prognostic value of CD47. In contrast, Gomez‐Llobell et al. [29] conducted a review of immune checkpoint inhibitors in acute myeloid leukemia (AML), reporting modest response rates and survival benefits, which underscores the need for further investigation into the role of CD47 in different malignancies. This finding is consistent with the results of previous studies that have identified CD47 as a critical factor in tumor progression and immune evasion. For example, a study conducted by Kosaka et al. reported that CD47 was highly expressed in 36.7% of breast cancer specimens, indicating its potential as a therapeutic target in this malignancy [30]. Moreover, CD47 has been linked to the epithelial‐mesenchymal transition (EMT), a process associated with increased invasiveness and metastasis in triple‐negative breast cancer (TNBC) [31]. Furthermore, the overexpression of CD47 has been documented in various malignancies, including colorectal and ovarian cancers [32, 33], and has been associated with adverse clinical features such as advanced tumor stage and poor differentiation. Our analysis corroborates these findings, revealing that high CD47 expression is significantly linked to advanced clinical stages and poor tumor differentiation, thereby emphasizing its role as a prognostic indicator. However, other investigations have yielded conflicting results. For example, Kim et al. [18] and Sercan et al. [13] found that CD47 did not demonstrate prognostic significance in studies on hepatocellular carcinoma and endometrial cancer. The discrepancies observed may arise from variations in study populations, methodologies, and the specific cancer types analyzed. It is noteworthy that our meta‐analysis included a diverse range of solid tumors, which may contribute to the robustness of our conclusions regarding CD4's role as a negative prognostic factor.

The mechanisms by which CD47 overexpression results in a poor prognosis are complex and multifaceted. CD47 functions as a “don't eat me” signal that inhibits phagocytosis by macrophages through its interaction with SIRPα [34, 35]. This interaction allows tumor cells to evade immune surveillance and also promotes the formation of an immunosuppressive tumor microenvironment, which facilitates tumor growth and metastasis [30, 36]. Moreover, CD47 has been linked to the induction of EMT, a process associated with increased invasiveness and stemness in cancer cells [37]. Given its capacity to regulate immune responses and foster tumor aggressiveness, CD47 is a pivotal factor in cancer progression. In addition to its role in immune evasion, CD47 expression has been linked to various clinicopathological features, including tumor differentiation, lymph node metastasis, and TNM staging [38, 39]. Our analysis demonstrated that elevated CD47 levels were markedly correlated with advanced clinical stages and poor differentiation, thereby substantiating its potential as a biomarker for stratifying patients based on prognosis. Nevertheless, it is crucial to acknowledge that although CD47 expression was not significantly correlated with sex or distant metastasis, the implications of these findings necessitate further investigation to elucidate the underlying biological mechanisms.

While our study has several notable strengths, including a comprehensive analysis of a large patient cohort and the use of robust statistical methods, it is important to acknowledge certain limitations. The observed heterogeneity in multivariate analyses suggests that factors such as tumor type, geographic location, and detection methods for CD47 may exert an influence on the outcomes. Moreover, the inconsistency in sample sizes, detection methods, and threshold values across the included studies may introduce biases that could affect the generalizability of our findings. It is recommended that future research aim to standardize CD47 detection methods and explore its role in combination with other biomarkers in order to enhance prognostic accuracy.

In conclusion, our meta‐analysis indicates that CD47 overexpression is significantly associated with poor clinical outcomes, advanced clinical stages, and poor differentiation in solid tumor patients, particularly, in cases involving tumors in the digestive and respiratory systems. These findings suggest that CD47 could serve as a valuable prognostic biomarker and therapeutic target. However, due to the limitations of this study, further validation through large‐scale clinical randomized controlled trials is necessary.

## Author Contributions

Y.Y., M.C., F.J., and S.M. conducted research design, literature search, data collection, and organization. Y.Y., Z.C., X.W., and Q.H. performed data analysis, interpretation, and manuscript writing. X.L. made a contribution to the guidance and revision.

## Ethics Statement

The authors have nothing to report.

## Conflicts of Interest

The authors declare no conflicts of interest.

## Supporting information


**Table S1:** Association of CD47 expression and smoke exposure.

## Data Availability

The data that support the findings of this study are available from the corresponding author upon reasonable request.
