# Supplementary material for: Survival and Clinicopathological Significance of CD47 in Human Solid Tumors: An Updated Systematic Reviews and Meta‐Analysis
Source: Cancer Rep (Hoboken). 2025 Aug 5;8(8):e70296. doi: 10.1002/cnr2.70296 (PMC12324951; doi:10.1002/cnr2.70296)
Supplement: Supplementary file 1 — Table S1: Association of CD47 expression and smoke exposure. [file CNR2-8-e70296-s001.docx]

**Supplement table 1. Association of CD47 expression and smoke exposure**.

| **Clinicopathological parameter**  **Smoke exposure (+ vs. - )** | **No. of patients** | **Cancer** **type** | **OR (95% CI)** | **Pvalue** | **Heterogeneity** | |
| --- | --- | --- | --- | --- | --- | --- |
|  |  |  |  |  | **I^2^ (%)** | **P‑value** |
| **Studies** | - | - | - | - | - | - |
| Arrieta et al(3) ,2020 | 169 | NSCLC | 1.19 (0.64, 2.21) | 0.5744 | - | - |
| Mario et al(2) ,2021 | 51 | LNET | 0.81 (0.23, 2.83) | 0.7434 | - | - |
| Xu et al(4),2020 | 191 | NSCLC | 0.32 (0.17, 0.60) | 0.0004 | - | - |
| Yang et al(11),2019 | 148 | PSC | 1.17 (0.58, 2.36) | 0.6685 | - | - |
| **Total** | 559 |  | 0.75 (0.53, 1.08) | 0.12 | 72 | 0.01 |

*Abbreviations:* NSCLC, nonsmall cell lung cancer; LNET, lung neuroendocrine tumors; PSC, pulmonary sarcomatous carcinoma;

OR, odds ratio.
